# Supplementary material for: The Colombian Zika Virus Isolate (COL345Si) Replicates in Prostate Adenocarcinoma Cells and Modulates the Antiviral Response
Source: Microorganisms. 2022 Dec 7;10(12):2420. doi: 10.3390/microorganisms10122420 (PMC9782197; doi:10.3390/microorganisms10122420)
Supplement: Supplementary file 1 [file microorganisms-10-02420-s001.zip › microorganisms-1914728-supplementary.pdf]

Supplementary Materials

Strains	Gen Bank	Lineage	Codon position																							
			80	106	107	123	130	139	151	691	763	982	1059	1118	1274	1477		2086			2611	2634	3213	3353	3392	3403
			C	C	C *	prM	prM	prM	prM	E	E	NS1	NS1	NS1	NS2A	NS2A	NS2B	NS3	NS4A	NS4B	NS5	NS5	NS5	NS5	NS5	NS5
ZIKV COL345Si	MH179341.1	Asian	I80	A160	D107E *	V123A	N130S	S139N	M151L	H691	V763M	A982V	K1059	R1118W	P1274L	A1477T		Y2086H			A2611	M2634V	T3213	T3353A	M3392V	V3403M
ZIKV (Meta-Colombia)	MH544701.1	Asian	I80	A160	D107	V123A	N130S	S139N	M151L	H691Y	V763M	A982V	K1059E	R1118W	P1274L	A1477T		Y2086H			A2611	M2634V	T3213I	T3353A	M3392V	V3403M
ZIKV/ Puerto Rico PRVABC59	KJ501215.1	Asian	I80T	A106T	D107	V123A	N130S	S139N	M151L	H691	V763M	A982V	K1059	R1118	P1274L	A1477T		Y2086H			A2611V	M2634V	T3213	T3353	M3392V	V3403M
ZIKV Cambodia	KU955593.1	Asian	I80	A160	D107	V123	N130	S139	M151	H691	V763	A982	K1059	R1118	P1274	A1477		Y2086			A2611	M2634	T3213	T3353	M3392	V3403

Figure S1. Identification of the mutations of a Colombian Zika virus isolate. Asterisks indicate the capsid protein mutation, C: D107E

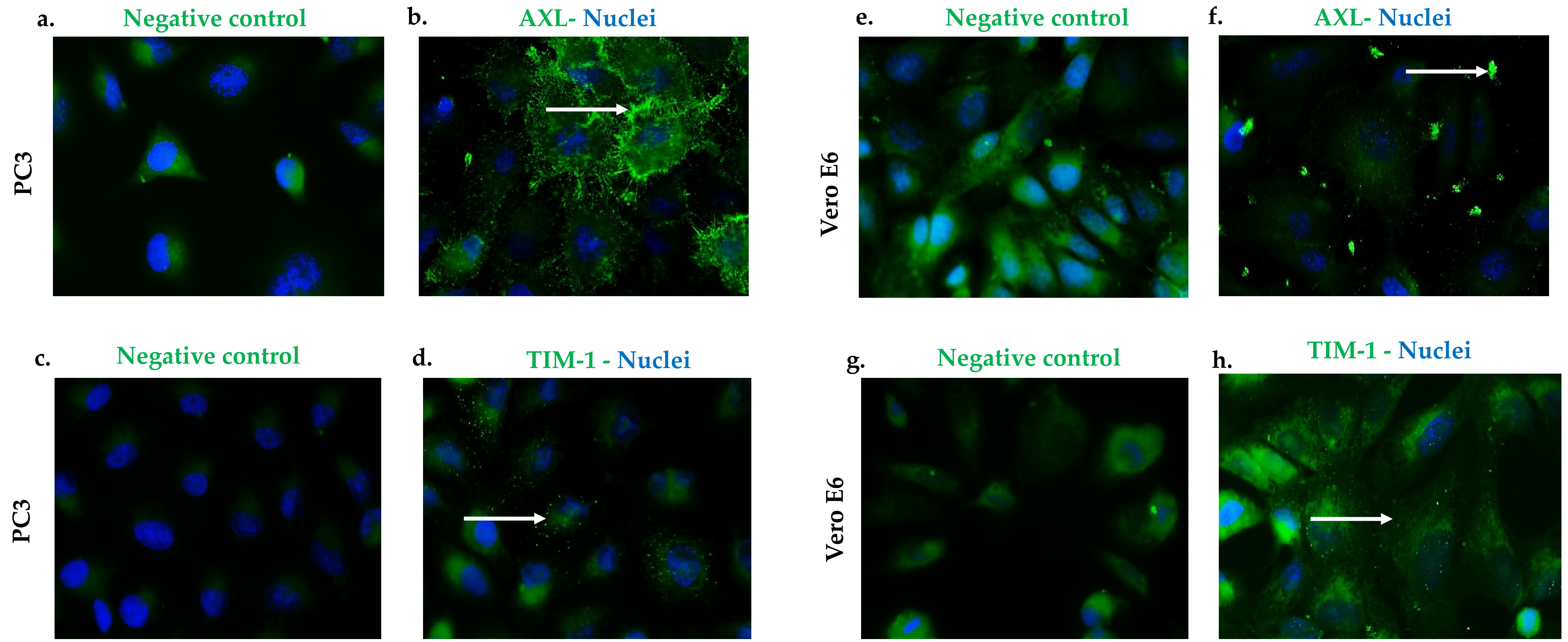

Figure S2. PC3 and Vero E6 cells express AXL and TIM-1 cell receptors. Pictures were captured at a 60× magnification. The photographs represent the subcellular location of AXL (**b**,**f**) and TIM-1 (**d**,**h**), and they correspond to three independent triplicate experiments. AXL and TIM-1 (green), and nuclei (blue). Figures **a**, **c**, **e**, and **g** are cells antibody secondary labeled and correspond to the negative controls

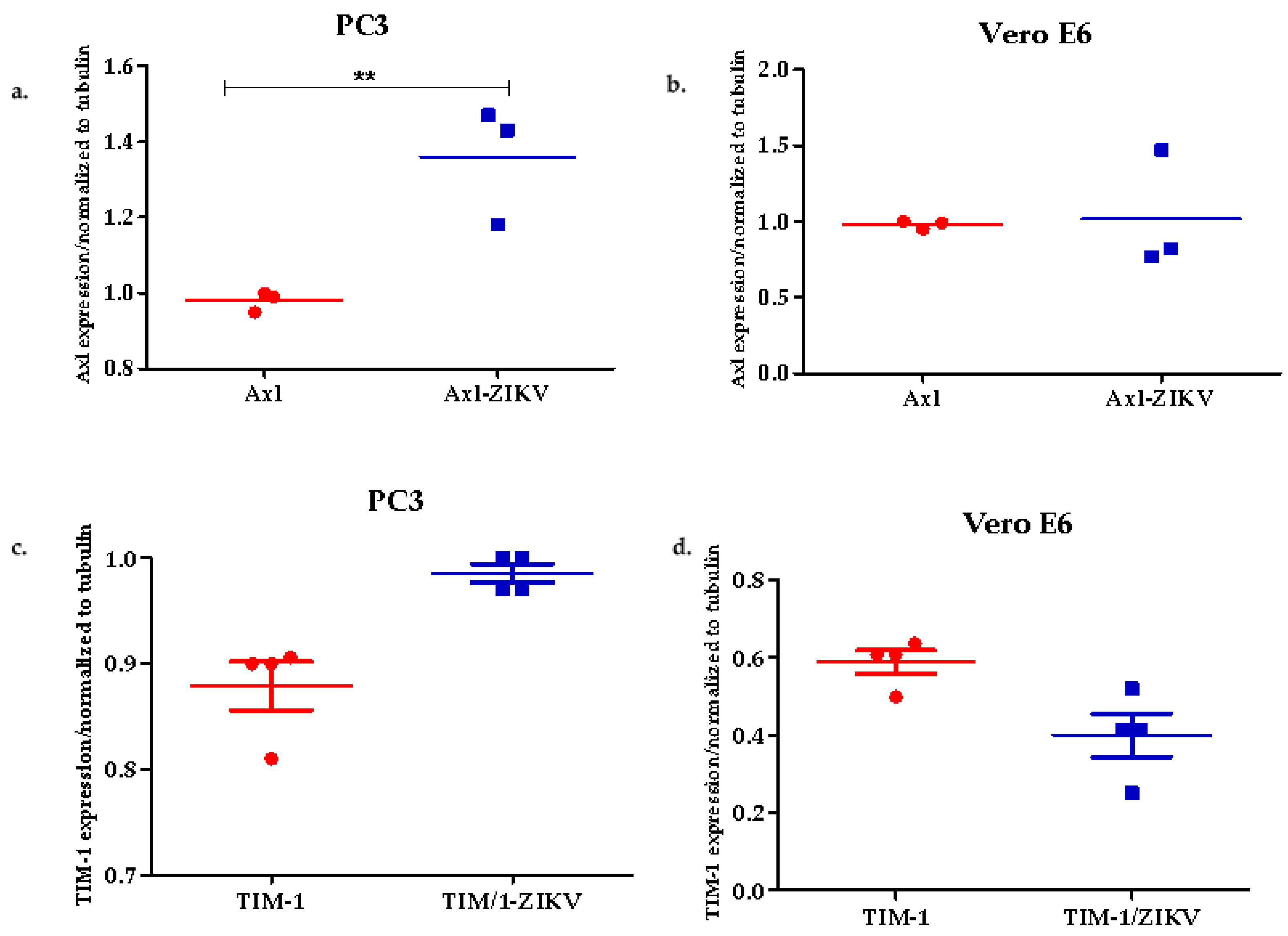

Figure S3. Detection of the AXL and TIM-1 receptors on infected and uninfected PC3 (MOI=3) by 24 h (**a**-**c**) and Vero E6 (**b**-**d**) cells through In-Cell Western.

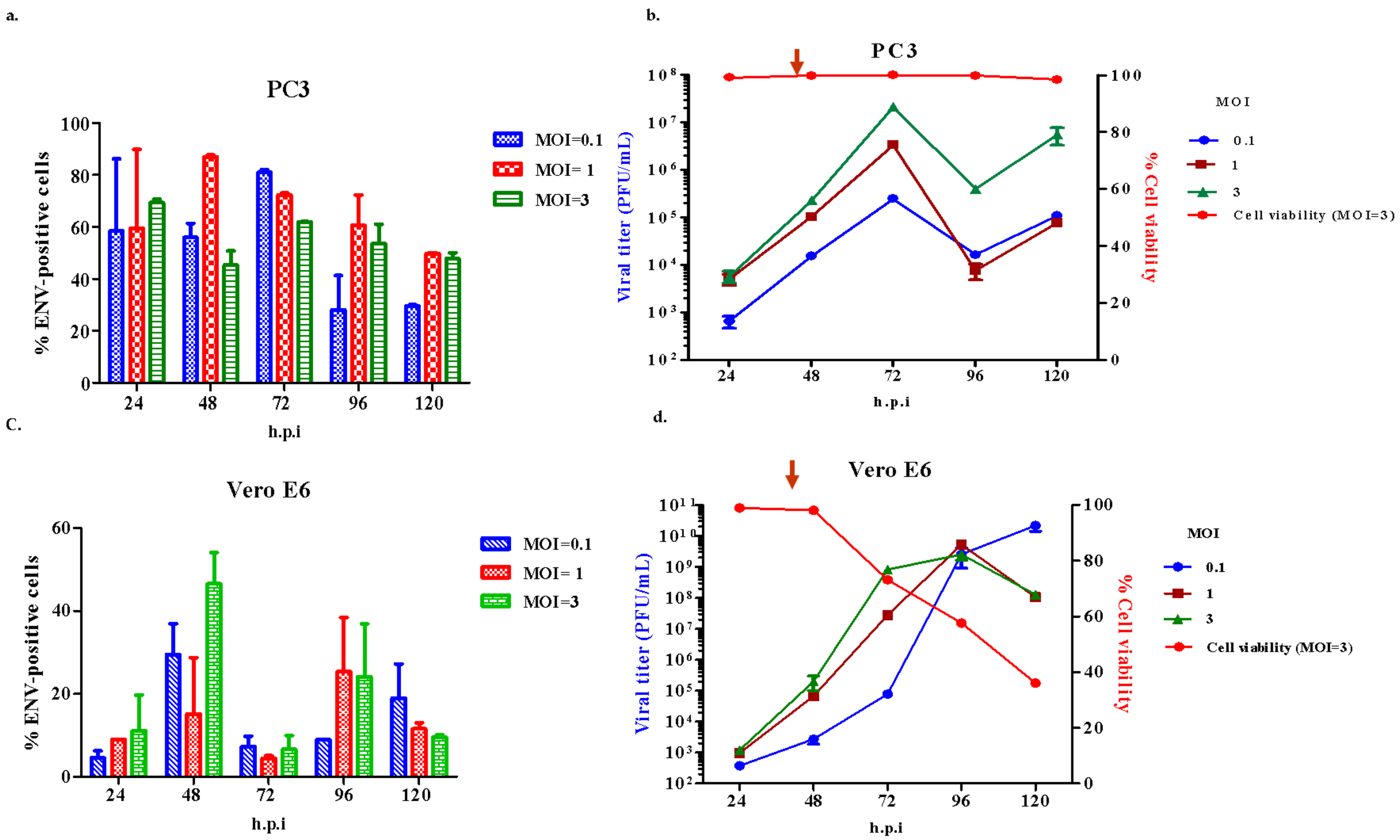

Figure S4. Zika virus replicates in PC3 and Vero E6 cells. The graphic represents the percentage of the viral envelope protein and plaque forming units viral on PC3 (**a**-**b**) and Vero E6 (**c**- **d**) cells infected with MOI= 0.1, 1, and 3

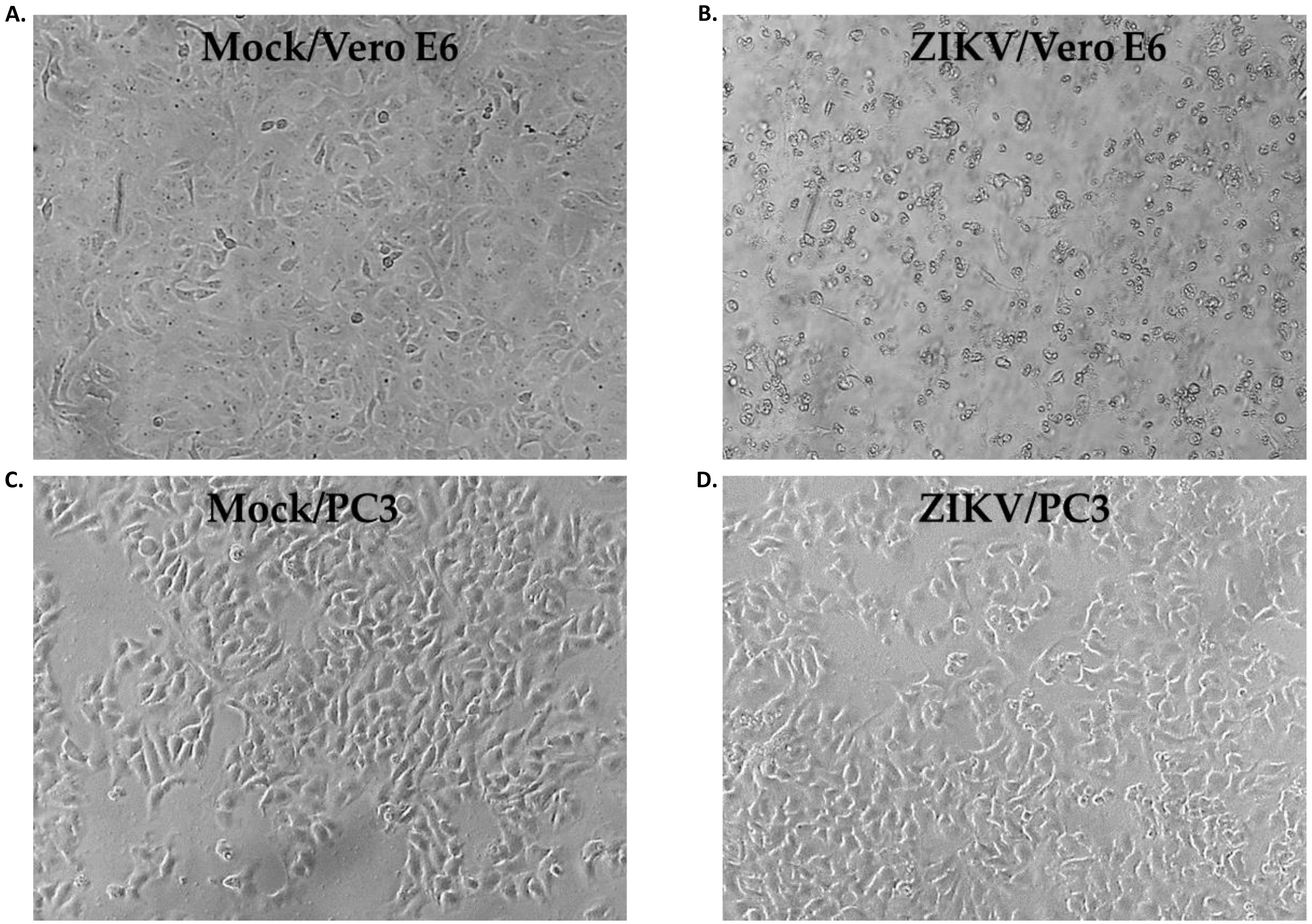

Figure S5. Cytopathic effects Vero E6 y PC3 cell lines during ZIKV infection. Cytopathic effects on Vero E6 (**b**) infected with ZIKV consisting of cell rounding and detachment at 5 days- post-infection. ZIKV did not cause cytopathic effects in PC3 cells (**d**).

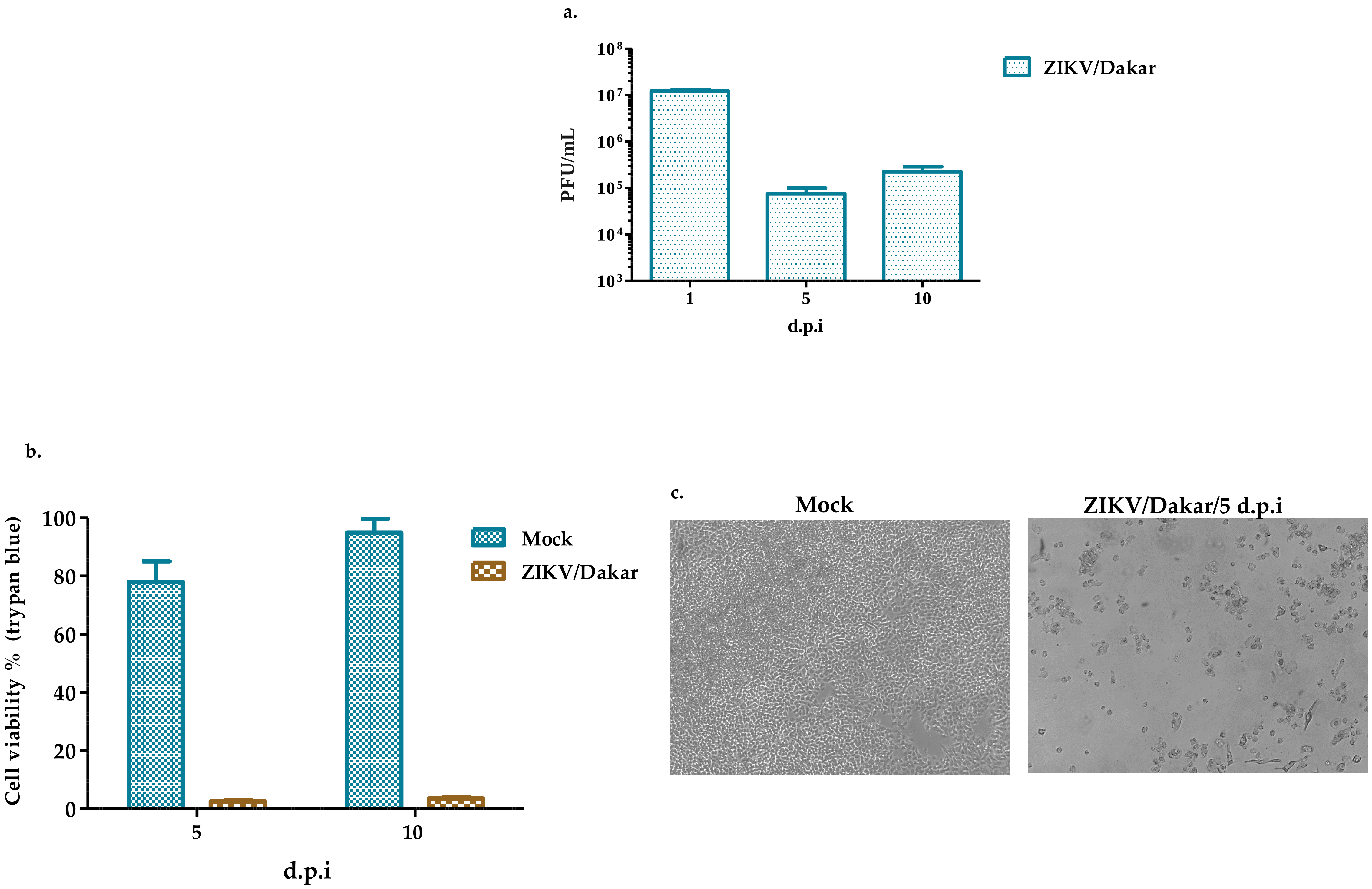

Figure S6. Zika- Dakar Virus replication (a) produces a cytopathic effect in the PC3 cells at 5 or 10 post-infection days (d.p.i) **(b-c).** Figure **b** shows the most representative picture.
